# Recent Advances in Microfluidic Paper-Based Analytical Devices toward High-Throughput Screening

**DOI:** 10.3390/molecules25132970

**Published:** 2020-06-28

**Authors:** Siraprapa Boobphahom, Mai Nguyet Ly, Veasna Soum, Nayoon Pyun, Oh-Sun Kwon, Nadnudda Rodthongkum, Kwanwoo Shin

**Affiliations:** 1 Metallurgy and Materials Science Research Institute, Chulalongkorn University, Soi Chula 12, Phayathai Road, Pathumwan, Bangkok 10330, Thailand; s.boobphahom@gmail.com; 2 Department of Chemistry and Institute of Biological Interfaces, Sogang University, Seoul 04107, Korea; nguyetmai.ly@outlook.com (M.N.L.); veasna_soum@yahoo.com (V.S.); vusskdbs@naver.com (N.P.); oskwon@sogang.ac.kr (O.-S.K.)

**Keywords:** microfluidic paper-based analytical devices, immunoassay, biomarkers, infectious diseases, drug analysis, environmental monitoring, food quality control

## Abstract

Microfluidic paper-based analytical devices (µPADs) have become promising tools offering various analytical applications for chemical and biological assays at the point-of-care (POC). Compared to traditional microfluidic devices, µPADs offer notable advantages; they are cost-effective, easily fabricated, disposable, and portable. Because of our better understanding and advanced engineering of µPADs, multistep assays, high detection sensitivity, and rapid result readout have become possible, and recently developed µPADs have gained extensive interest in parallel analyses to detect biomarkers of interest. In this review, we focus on recent developments in order to achieve µPADs with high-throughput capability. We discuss existing fabrication techniques and designs, and we introduce and discuss current detection methods and their applications to multiplexed detection assays in relation to clinical diagnosis, drug analysis and screening, environmental monitoring, and food and beverage quality control. A summary with future perspectives for µPADs is also presented.

## 1. Introduction

Microfluidics is the science of fluid manipulation in a microscale network and deals with controlling fluid samples with low volumes (10^−9^ to 10^−18^ L) inside micrometer-scale channels [1,2]. Though the first known microfluidic device was documented in 1979 [3], it mostly dealt with materials like silicon, glass, and polymers such as polymethylmethacrylate (PMMA), polystyrene (PS), polycarbonate (PC), and polydimethylsiloxane (PDMS) [4]. Miniaturization of experimental apparatuses has led to lab-on-chip environments in which sample and reagent consumption is low, reactions are faster, and mixing or separation is more efficient, thereby enabling quick, modern, high-technology research [5]. Hence, microfluidics has garnered significant attention in analytical chemistry.

After a few decades of development, microfluidics started to blossom with the utilization of papers (chromatography, filter, and nitrocellulose paper); this was triggered by Whitesides’ publication in 2007 [6]. These inexpensive and ubiquitous materials allow researchers to use simple and feasible methods with minimal accessories to create affordable microfluidic paper-based analytical devices (µPADs) for point-of-care (POC) application in clinical diagnostics [7,8,9,10], environmental monitoring [11,12,13], and food analysis [14,15,16]. Conventional µPADs transport fluid samples and reagents in a continuous-flow way, which can be defined as paper-based continuous-flow microfluidic (p-CMF) devices. On the other hand, the term digital microfluidic (DMF), which was first introduced in 2000 by Pollack and Fair, is to describe devices in which individual droplets are transported without valves or channels. This concept became more practical by lowering the applied voltage as shown by Kim’s group [17]. To leverage the use of paper, Shin’s group [18] and Wheeler’s group [19] introduced paper-based DMF (p-DMF) devices in 2014 as digitally controlled microfluidic platforms for cost-effective and programmable analytical assays. Owing to intrinsic attractive features of DMF, p-DMF devices render transporting, mixing, merging, spitting, dispensing droplet(s), and delaying the actuations that are the most important basic droplet manipulations in protocols for performing a specific microfluidic assay. Together, both p-CMF and p-DMF devices can serve as portable and disposable platforms that once more functionalities are added, such as storing reagents, patterning electrodes, integrating with other sensors, e.g., electrochemical sensors, they will complete prerequisite characteristics for analytical assay [5,20,21].

In fact, according to our SciFinder search of journals, patents, reviews, conference abstracts, dissertations, editorials, and letters at the time of this writing, 2071 reports specifically mentioned “paper-based” and “microfluidic” and 831 reports mentioned “paper-based analytical device”. With their many advantages, µPADs are most suitable for use in point-of-care testing (POCT) and field monitoring, for which high-throughput screening is often required. For example, on-site rapid detection and high-throughput screening for infectious diseases, such as MERS, SARS, and SARS-CoV-2, may reduce the possibility of a disease outbreak. µPADs using different isothermal amplification techniques were shown to have a capability for high-throughput nucleic acid testing [22,23]. In different areas, high-throughput screening might have specific definitions. However, here, we define it as fast screening on a relatively large scale, either with multiple samples or with one sample, but with multiplex replicates or simultaneous measurements of different factors. If µPADs are to achieve this ultimate purpose, they must be capable of being integrated with supporting features, including high-throughput sample preparation (and separation, in certain cases), high-throughput sample handling, and finally, high-throughput readouts and data acquisitions.

**Figure 1:**
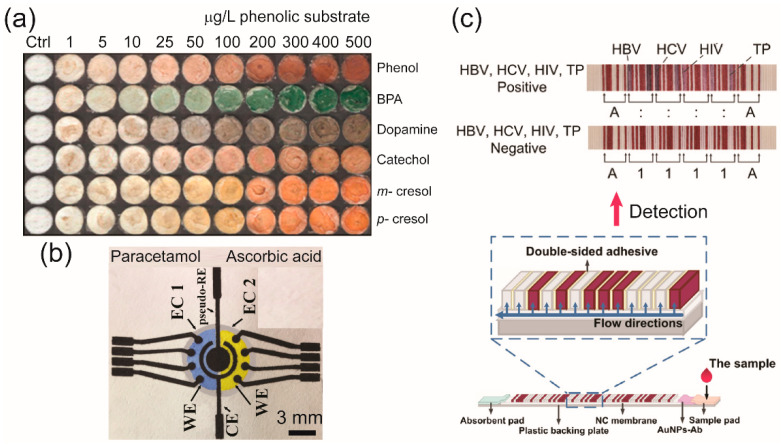
Represented microfluidic paper-based analytical devices (µPADs) for high-throughput screening. (**a**) The μPADs for the colorimetric detection of phenolic compounds. Reproduced with permission from the authors of [24]. Copyright 2012, American Chemical Society. (**b**) The electrochemical (e)-μPADs for multiplexed analytical detections of paracetamol, caffeine, and ascorbic acid in drugs. Reproduced with permission from the authors of [25]. Copyright 2018, Elsevier B.V. (**c**) Lateral flow assay (LFA) that can be used for detecting four biomarkers (hepatitis B virus (HBV), hepatitis C virus (HCV), human immunodeficiency virus (HIV), and Treponema pallidum (TP)), simultaneously. Reproduced with permission from the authors of [26]. Copyright 2017 under the terms and conditions of the Creative Commons Attribution.

In this review, we summarize recent advances in paper-based microfluidic devices that have been claimed to be successful or have the potential to be applied for high-throughput screening. We will, firstly, go through the methods for fabricating µPADs, and the techniques used to improve the performances of μPADs, with a focus on high-throughput readouts and data acquisitions. Last, but not least, we discuss current detection methods and applications related to the multiplexed detection ability of μPADs in many research areas. Additionally, to explore potential novel research directions, we discuss current limitations and future perspectives of μPADs. Represented µPADs for high-throughput screening are depicted in Figure 1.

## 2. Fabrication of µPADs

### 2.1. Paper-Based Dontinuous-Flow Microfluidics Devices

We describe fabrication strategies highlighting novel techniques and on how different research groups have successfully oriented their devices toward high-throughput screening, including rapid fabrication, mass production (with high reproducibility), rapid prototyping, and compatibility with automated processes. We summarize the fabrication methods of p-CMF devices, integration of functionality and reproducibility, and mass production automation fabrication processes.

#### 2.1.1. Methods for Rapid Fabrication

From the very beginning, µPADs have been dominantly fabricated by using simple, rapid methods that facilitate prototyping and minimize cost while ensuring high-quality testing. This coincides with the trend to fabricate general microfluidic devices based on the cost of materials and related equipment and tools, faster turnaround time, and increased functionality [27]. Table 1 shows a collection of reported methods used for the quick fabrication of µPADs. For an explanation of each fabrication method and for the procedures used in each method, readers are redirected to excellent review papers [28,29,30,31].

#### 2.1.2. Integrated Functionalities

The need to apply research to solve real-life issues has led to the need to add more functions to µPADs to fulfill the testing requirements, those requirements ranging from 3D-guided channels for fluid samples transportation, sample preparation, programming fluid transportation, and sensing materials incorporation for the readout of results.

**Figure 2:**
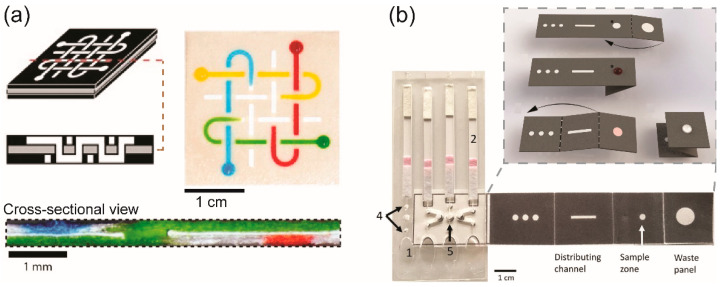
Representative methods for the fabrication of 3D-µPADs. (**a**) 3D-µPAD was fabricated by stacking layers of paper channels and adhesive tapes to form 3D channels. Reproduced with permission from the authors of [58]. Copyright 2008, The National Academy of Sciences of the USA. (**b**) 3D-µPAD was fabricated by using an origami method to form a 3D channel. The channels were folded that allowed fluid samples to flow in a vertical direction. Reproduced with permission from the authors of [86]. Copyright 2019 under the terms and conditions of the Creative Commons Attribution.

Herein, we pay more attention to 3D-µPADs. As proposed by Whitesides’ group, 3D-µPADs allow transport of fluids both laterally and vertically, enabling distribution, combination, and/or filtration of fluid to perform a function or to add a new function to the system [58] (Figure 2a). They are especially suitable for high-throughput screening in terms of our definition. Compared to the channels in 2D-µPADs, those in 3D-µPADs offer higher flow speed and more capability to perform multistep chemical reactions orderly or multiple assays simultaneously [84]. For instance, to the best of our knowledge, a 3D origami-based µPAD, which had been designed to extract nucleic acids from viscous samples, was first reported by Govindarajan et al. in 2011 [85]. A year later, Ge et al. reported a multifunction-integrated immunodevice based on a similar method; that device contained (i) a test tab surrounded by (ii) a filter tab to obtain serum from treated whole blood samples by separating red blood cells, (iii) two waste tabs for washing off nonspecifically bound proteins, and (iv) a reagent tab for the distribution of chemiluminescence reagents to obtain a detectable signal. When these tabs were folded correctly, multiplex immunoassays could be completed within 16 min [60]. Figure 2b shows a representative of a 3D-µPAD fabricated by using an origami method and 3D-µPAD was designed specifically for assay detection of DNAs [86].

Sample preparations including separation and preconcentration of analytes and the mixing of reagents are very important steps in analytical assay protocols. In µPADs, preconcentrating can be done by using the electrophoretic separation technique where mainly two conductive electrodes (anode and cathode) are integrated into a p-CMF channel. The electrodes located at the beginning and the end of 2D or 3D (origami-based) paper-based channels are applied with a certain electric field to separate the target analytes from the mixture based on the interaction between the analytes and electrophoretic force [87,88,89]. When the electrophoretic separation technique is integrated into µPADs, it enables users to analyze a sample that has relatively low concentration and to improve the limit of detection (LOD) [88,90,91]. This technique was applied successfully in µPADs to preconcentrate various biomarkers including amino acid [92], bovine serum albumin (BSA) [93,94], DNA [95], and microvesicle/exosome [96]. Electrophoretic separation technique has been used in µPADs as a sample preparation step in various analytical detection methods such as ion-transmission mass spectrometry (MS) for analysis of amino acids [97], matrix-assisted laser desorption ionization time-of-flight mass spectrometry (MALDI-TOF-MS) for detection of albumin and glycated hemoglobin (HBA1c) [98], and lateral flow immunoassay (LFA) for the detection of IgG [90]

**Figure 3:**
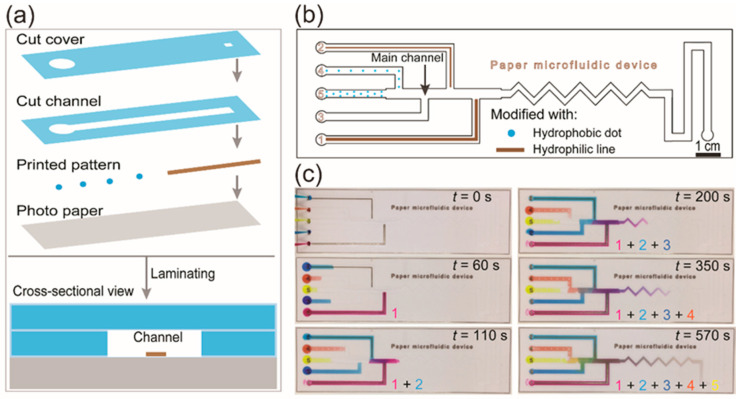
(**a**) Schematic illustration of fabrication and (**b**) programming fluid transport in a µPAD, respectively. (**c**) Fluid transport in microchannels was controlled quantitatively by using printed hydrophilic and hydrophobic patterns. Reprinted with permission from the authors of [99]. Copyright 2020, The Royal Society of Chemistry.

Typically, fluid control and programmability are vital to automate and to minimize the processing in the fabrication and the operation, but maximize the use of µPADs. By controlling the fluid flow in the µPAD, researchers have been able to achieve high-sensitivity detection and automated programmable assays [99,100]. Because fluid transport in the paper-based channel is induced by the capillary action generated by the interfacial interaction between the fluid and the fibrous network of the paper channel, the flow of fluid can be controlled by changing the surface energy, permeability, dimensions, and geometry of the paper channel [101,102,103,104,105,106]. Commonly, those paper channels are made of chromatography paper and filter papers. For the first time, Soum et al. introduced programmable photo-paper-based microfluidic devices in which the fluid flow could be quantitatively controlled. Fluid flow in the photo-paper-based channel can be controlled by using hydrophobic and hydrophilic patterns to decrease and increase the speed, respectively. Depending on the shapes and width of the patterns modified in the channel, the fluid transportation can be controlled quantitatively (Figure 3) [99]. Recent review papers have summarized progress in the development of flow control tools and the integration of those tools for paper microfluidics [30,31,107,108].

To detect the target analytes, the paper-based microfluidic platforms must be integrated with sensing materials such as reagents (chemicals, enzymes, antibodies) or electrochemical sensors to provide detectable signals such as color and electrical current. These sensing materials can be stored directly by physical absorption in the matrix of the paper and chemically immobilization on the fibrous paper while electrochemical sensors can be patterned on the surface of the paper. Being able to store reagents, µPADs make processes of analytical assays become faster and easier with low reagent consumption because less equipment and steps are involved. However, the stability of the reagents is lower compared to those reagents stored in the standard condition in the laboratories. To provide a longer lifetime of the reagents stored in the µPADs, researchers need to incorporate with functional materials for stabilization purposes and identify optimum storage conditions such as temperature, humidity, and light [20]. Electrochemical sensors can be printed directly on the surface of paper being used for µPADs by using screen printing, inkjet printing, and ballpoint pen printing methods. Screen printing is the most suitable method for printing electrochemical sensors on chromatography or filter paper because the method can print high-viscosity (500–50,000 cP) conductive inks that generate conductive electrodes within a single printing time [40]. The inkjet printing may require several or single printing time(s) to pattern good conductive electrodes because it can only print low viscosity ink (1–40 cP), which depends on the type of paper and inkjet printer used for the fabrication [109,110]. Lastly, the ballpoint pen printing method was reported that it could print conductive electrodes on photo paper within a single printing time [111,112]. A recent review paper by Noviana et al. has summarized methods for the fabrication of electrochemical paper-based devices [21].

#### 2.1.3. Reproducibility and Mass Production

Reproducibility, which we limit to the reproducibility of results, is defined as the ability to perform and confirm a previously reported finding by using procedures that are the same or closely matched to those in the original setting [113]. It is an important criterion in assessing the fabrication and the performance of a device. For robust results, during prototyping, device components and experiment parameters must be optimized if an efficient µPAD is to be produced. For this, a simple method will facilitate the process. Garcia et al. presented a handheld stamping process that produced a µPAD in seconds independently of the user’s technical skills [114]. We considered the fabrication methods that involve digitally control (e.g., wax printing, inkjet printing, and digital cutter) provides high reproducibility compared to the manual control methods (e.g., handwriting) [18,33,34,36,37,53,54,73,115].

When addressing fabrication, one must consider the mass production and the abilities to automate and/or program a device; these factors have a closed relationship and affect one another. Mass production uses a standardized process for creating interchangeable parts in large quantities at a low cost [116]. Normally, for a product to be produced in large quantities, (i) it should have a useful proven application and an economic value, (ii) it can be manufactured with minimal, cost-effective equipment, tools and materials, and (iii) it should be fabricated or handled via automation with less power consumption. Akyazi et al. provided a critical review that analyzed the strengths and weaknesses of each fabrication method and the potential to apply those methods commercially [117]. Due to isotropic wicking and multidirectional transport of fluids in the paper, the authors strongly emphasized that for successful commercialization of µPADs, an integrated flow control method was needed. We appreciate this work albeit the fact that objectively, no perfect fabrication method exists, particularly in this new exploding field. In summary, the cost and the value of the final product must be considered when selecting and optimizing µPADs for use in specific applications.

Wax screen printing, as an example, is reported to be a possible fabrication method for mass production [118]; however, it requires a heating step, and wax materials cannot be used with any organic solvents. Sameenoi et al., therefore, proposed a one-step polymer screen printing method using polystyrene, which allowed mass production of flexible µPADs in a roll-to-roll format [50]. The demonstration showed their devices had good analysis capability for both inorganic (hydrogen peroxide–H_2_O_2_) and organic (2,2-diphenyl-1-picrylhydrazyl–DPPH) solvents. Another example is the use of a commercial inkjet printer and an XYZ dispensing platform to mass-produce barcoded µPADs with high precision [36]. The fabricated devices showed good performance for rapid multiplexed immunoassays and nucleic acid detection. Based on our own perspective, mass production of paper-based microfluidic devices can be achieved when a fabrication method for μPADs is transferred to a manufacturing process after it is well optimized in the laboratory. However, the cost of material for the fabrication should be a primary concern before shifting to mass production.

### 2.2. Paper-Based Digital Microfluidic Devices

In p-DMF devices, the movement of a fluid droplet is induced by using an electric field on the electrode arrays that are coated by a hydrophobic dielectric layer. The droplet can be actuated by electrowetting force created at the interface between the hydrophobic dielectric layer and conductive droplet. As the applied voltage increases, the contact angle of the droplet becomes smaller as the wettability is increased [119]. This property can be explained by the Young-Lippmann equation:

$$\cos\theta_{L}\left(V\right)=\cos\theta_{Y}\left(0\right)+\frac{CV^{2}}{2\gamma_{lv}}$$

where *θ_L_*(*V*) and *θ_Y_*(0) are the contact angle of the droplet when a voltage *(V)* and no voltage are applied, respectively. *C* and *V* are the capacitance, and applied voltage, respectively. *γ_lv_* is the interfacial tension of the droplet.

**Figure 4:**
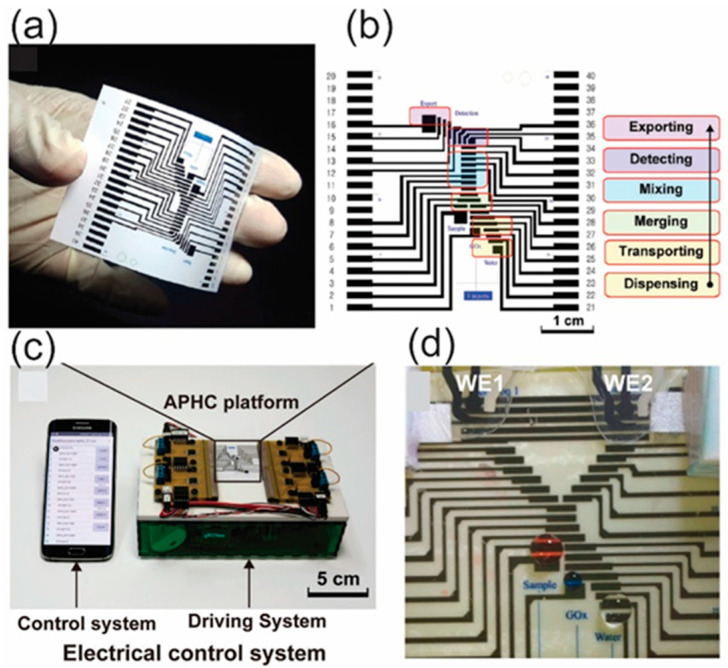
(**a**) A photograph of a p-DMF device, (**b**) schematic illustration fluid handling on the device, (**c**) a controlling system for operation of the p-DMF device, and (**d**) operation of the device to transport droplets. Reproduced with permission from the authors of [78]. Copyright 2017, WILEY-VCH Verlag GmbH & Co. KGaA, Weinheim.

Two main components for the fabrication of a p-DMF device are conductive electrode arrays and hydrophobic dielectric layers that have the function to deliver applied voltage to actuate electrodes and to suppress electrical current from leaking, respectively. Moreover, an electrical switching system (operation system) to activate each of the individual electrodes is required for the basic droplet manipulations of p-DMF devices. The inkjet method, a familiar printing method frequently used for handling fluids through EWOD, allows electrodes to be printed on paper in a very accessible way by using personal computers and printers (Figure 4a) [78]. On the printed electrode, as mentioned above, is a dielectric layer for preventing electrolysis; a parylene-C coating fabricated using chemical vapor deposition (CVD) is commonly coated with a hydrophobic layer that reduces the contact angle and increases the initial contact angle. These surface modification steps cause the fluid to move smoothly. To efficiently program the chip design and to reduce its complexity, researchers have proposed that chips be constructed from component libraries and verified through simulations before fabrication [120]. By decomposing the system into modules, which are sets of components with customized functions, one can reduce the system’s complexity. The system can be controlled by using computer-based software and a smartphone app (Figure 4c,d) [78].

Fobel et al. printed five reservoir electrodes and 19 drive electrodes on photo paper by using inkjet printing with silver nanoparticles [19]. Through automation with an open-source DropBot drop controller, they performed the 63 steps (27 dispense, 18 mix, and 6 sprite steps, 12 measurements) required for a homogenous chemiluminescence assay with higher reproducibility in less than one hour with only the initial three pipettings. Meanwhile, Yafia et al. used a mask to squeeze a CNT ink onto paper to print the electrode by using screen printing [81]. In this case, complete contact between the screen and the paper substrate is important for resolution. Instead of materials such as parylene-C, polydimethylsiloxane (PDMS), and Cytop ™ amorphous fluoropolymer, which are mainly used as dielectric layers [121], applying a stretched parafilm, they were able to avoid the need for additional equipment. They also realized the automation of fluid routing by using an Arduino mega 2560 unit and a card edge connector (3-5147490-0, TE connectivity).

Another very simple method for printing electrodes is the ballpoint pen printing method [81]. By mounting a conductive ballpoint pen with a digital plotter, printing can be done quickly and very easily without expensive equipment. For the dielectric layer, the authors of Ref. [79] used a simple process of wrapping the electrodes with commercial premade linear-low-density polyethylene (LLDPE) plastic (17-uL thick), which is inexpensive and easy to obtain. Silicon oil was applied to the top and the bottom of the plastic wrap to avoid bubbles between the electrodes and the wrap and to reduce friction between the fluid and the chip. This significantly reduced the contact angle to facilitate fluid movement. Their control system for the chip consisted of a hardware prototype and a smartphone, and control was accomplished by running a custom-developed app that could send commands via Bluetooth. As the chip can be manufactured using a variety of easy ways and can be automated using electricity, bioassays and diagnoses can be performed quickly and conveniently with minimal manpower. A summary of methods for the fabrication of p-DMF devices is shown in Table 1.

## 3. Detection Techniques

Even though μPADs provide extraordinary advantages, including simple fabrication and mass-scalability at an affordable cost, if an advanced paper-based analytical device providing high performance and sensitivity is to be achieved, suitable detection mechanisms are necessary for the μPADs. The most common detection techniques in μPADs are colorimetric, electrochemical, chemiluminescent, and electrochemiluminescent techniques. Herein, we briefly summarize and compare such detection techniques.

### 3.1. Colorimetric Technique

**Figure 5:**
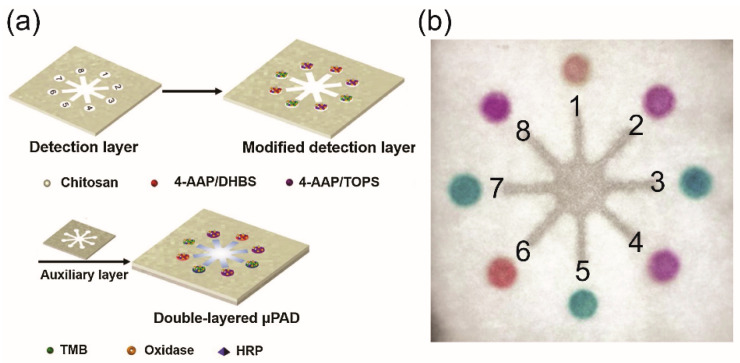
(**a**) Schematic illustration of the device structure, (**b**) color photograph of a μPAD after colorimetric detections of glucose (1, 2), uric acid ((3, 4), choline (5, 6), and lactate (7, 8). Reproduced with permission from the authors [125]. Copyright 2019, Elsevier B.V.

Colorimetric assays have been widely used in μPADs for the simultaneous detection of target analytes, which is usually related to a biological reaction or color-change chemical assay, and have provided qualitative or semiquantitative results [122]. The results can be visualized qualitatively and semiquantitatively by a naked eye due to a biological or chemical interaction (Figure 5). The first μPAD was demonstrated for the colorimetric detection of glucose and proteins and was based on a change in color when the sample was introduced into the reaction zone [6]. However, the detection sensitivity in conventional paper-based devices is relatively low because they suffer from limitations on the physical flow behavior and the time delay of the reaction. The use of metal and metal oxide nanoparticles [123,124], biological catalysts [125], and chitosan [126] to modify the paper’s surface has been proposed to enhance the detection signals from μPADs used for automated and multiplexed colorimetric analyses. In addition, the design of μPADs as multilayers and origami platforms can significantly improve the colorimetric signal for highly sensitive detection. Because the sample solution added onto multilayered μPADs can be automatically pretreated in the microfluidic channel before carrying out the colorimetric reactions at the detection zones, the color intensity and reproducibility are greatly increased [60,61,125,127]. Integrating colorimetric μPADs with cell culture microarrays has been developed for high-throughput drug screening. A μPAD containing a drug–concentration gradient was designed and fabricated for high-throughput testing of the cell’s response to drugs. This drug screening platform can be applied for drug discovery and for diagnostic studies with simultaneous and parallel tests of drugs under various concentration gradients [128].

Several conventional μPADs used for colorimetric detection provide low sensitivity of detection compared to UV–visible spectroscopy and absorption spectroscopy. They are mainly limited by the interpretation of color intensity via the naked eye, which is different for each individual, by the nature of the ambient light, and by the properties of the paper substrate [122]. Colorimetric μPADs require additional devices, such as a camera, scanner, and smartphone-based reporting system, to increase their accuracy, detection sensitivity, and high-throughput ability. The intensity of a color signal can be interpreted using imaging software, where a pixel value is related to the concentration of the analyte. The data can be analyzed by using such a system [129,130,131,132].

Remarkably, the p-DMF chip, which is governed by the same basic principle as other DMF devices involved with fluid manipulation by using EWOD. The p-DMF device is able to manipulate small-volume droplets individually without the channels, pumps, and microvalves required by other devices; hence, it is proper for multistep, as well as for other single-step, assay procedures [133,134]. Not only the p-DMF devices can transport fluids and delay fluid delivery, they also efficiently mix several samples with reagents for analysis and for tunable dilutions. Moreover, p-DMF devices are cost-effective, portable, and disposable, which makes them more suitable for point-of-care testing than other DMF devices on glass or polymer substrates.

**Figure 6:**
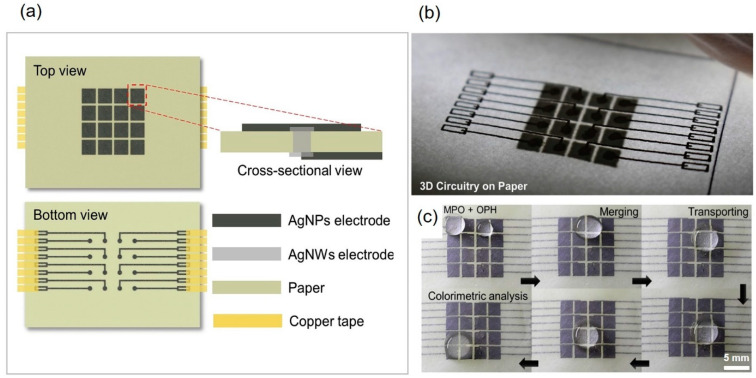
(**a**) Schematic illustration of the fabrication of two-dimensional electrode arrays for a p-DMF device, (**b**) photograph of the printed p-DMF device showing the 3D circuitry over an LED lamp, (**c**) the p-DMF device showing merging, mixing, and transport functions. Reproduced with permission from the authors of [82]. Copyright 2018, Elsevier B.V.

Recently, p-DMF devices have emerged as microfluidic platforms for colorimetric detection. Abadian et al., with the goal of expanding microfluidic capabilities for POCT, developed a new p-DMF device. In the detection, the device performed preprocessing steps of controlling the flow of the liquid sample and transferring it to a paper-based channel where the reagent was immobilized for glucose detection [80]. The detection performance of this device indicated that it could be applied as a POCT device in multistep microfluidics assays, such as a biosensor in enzyme-linked immunosorbent assays (ELISA). Jafry et al. demonstrated a novel p-DMF chip based on double-sided electrohydrodynamic jet printing of circuitry and the dispensing of high-viscosity silver nanoparticles as a two-dimensional array of electrodes on a single paper sheet. The proposed p-DMF device effectively performed as a low-cost, disposable POCT platform for pesticide detection (Figure 6) [82]. The p-DMF platform could be operated with a low operating voltage to move the droplet toward an actuated electrode; such a low operating voltage would not be harmful to bioassays and would be appropriate for portable POCT applications. Atabakhsh et al. introduced thermocapillary actuation working with low-voltage batteries to manipulate, transport, and confine the droplets on a p-DMF platform. This proposed device could enhance the controllability of water-droplet transport and decrease the droplet’s surface tension; it was applied for a biochemical glucose colorimetric detection assay [135].

Colorimetric detection has been widely employed in many μPAD designs because of the simplicity, ease of operation, handheld capability, and practical applications of μPADs. Nevertheless, colorimetric detection still suffers from the main drawbacks of low selectivity, low sensitivity, and longer detection time. In addition, additional tools, such as cameras or scanners, must be used to amplify the colorimetric signal, leading to more operational steps and higher analysis costs. μPADs using electrochemical detection have emerged as a promising alternative to high-sensitivity quantitative measurements, as will be discussed in detail below.

### 3.2. Electrochemical Detection

An electrochemical technique has become one of the most promising methods of analytical detection for μPADs because of the availability of relatively low-cost potentiostats, the ease of their use, and their easy miniaturization for on-site measurements. In contrast to colorimetric detection, this technique can provide both qualitative and quantitative information in the nanomolar range with high sensitivity and selectivity, enabling low detection limits and fast responses. Moreover, various material-modified electrodes on μPADs have led to increased surface areas and increased conductivities, which significantly improve the response signal of μPADs used for electrochemical detection. Therefore, the results from a number of research programs involving the development of electrochemical μPADs (e-μPADs) using metallic nanoparticles (Au, Ag, Pt NPs), carbon-based nanomaterials (graphene, CNTs), and chemical/biocatalysts (enzymes, antibodies) for analytical measurements of target analytes have been reported.

**Figure 7:**
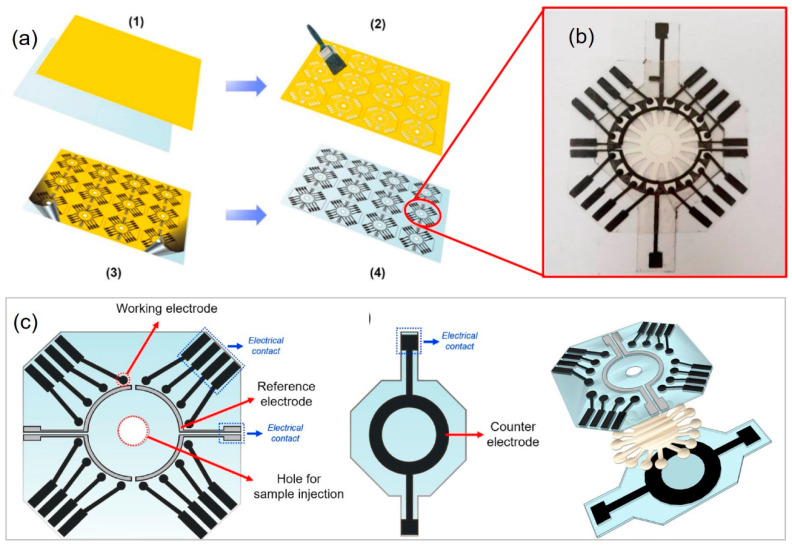
(**a**) Schematic representation of electrode construction, consisting of the following steps: (1) a vinyl adhesive is fixed onto a polyester sheet; (2) the adhesive is cut in the desired design, followed by the deposition of carbon conductive ink; (3) removal of the adhesive after the inks have dried; (4) a batch of electrodes ready to be assembled with the microfluidic device. (**b**) Photograph of an assembled 16-microfluidic channel platform. (**c**) Steps for device layer assembly consisting of the WEs/REs and CE layers’ design. Reproduced with permission from the authors of [137]. Copyright 2019, Elsevier B.V.

Currently, e-μPADs exhibit the possibility of performing multianalyses, which sets the stage for the development of various promising applications, especially in POCT and biomedical diagnosis. Wu et al. fabricated a multiplexed e-μPAD by using wax printing and screen printing for the simultaneous detection of multiple tumor biomarkers in a clinical sample. This design provided wide linearity and a low detection limit with a good correlation to the data obtained using standard equipment [136]. The design of e-μPAD platforms without a wax printer has been introduced as an alternative for simultaneous multiple-analyte determination. Fava et al. revealed the fabrication of a disposable e-μPAD using an adhesive mask containing 8–16 channels that could provide a multiplexing capability with a low detection limit of 3 × 10^−5^ mol L^−1^ for glucose detection in urine (Figure 7) [137]. Additionally, Fava et al. utilized 16 working electrodes on one e-μPAD for the multiplexed analyses of four biomarkers in real urine samples [138].

**Figure 8:**
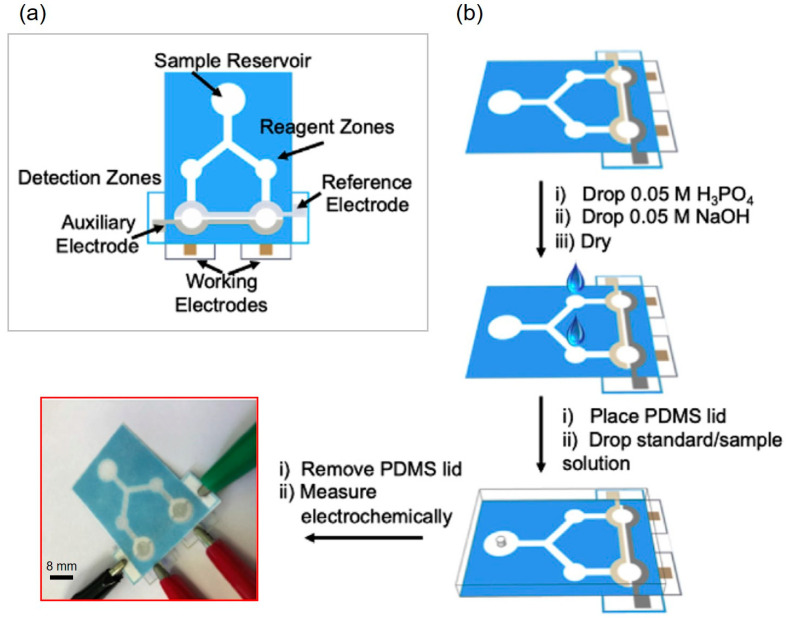
Schematic representation of a Janus e-μPAD: (**a**) design of the Janus e-μPAD and (**b**) operation for multiplexed detection with in situ pH adjustment. Reproduced with permission from the authors of [139]. Copyright 2019, Elsevier B.V.

Additionally, the simultaneous detection of multiple targeted analytes from a single sample by using electroanalytical techniques is challenging due to poor selectivity and sensitivity for other species in the mixture caused by changes in the assay conditions towards a target analyte. To overcome this issue, Henry’s group reported Janus e-μPADs as a new approach for high-performance multiplexed electrochemical sensing under different solution conditions on a single sample at the same time by using a single potentiostat. The Janus e-μPAD was utilized for the simultaneous detection of multiple neurotransmitters with the capability for in situ pH control and optimized electrochemical experiments. This device showed great potential as a low-cost platform that could provide multiplexed analysis by using simple steps (Figure 8) [139]. The Janus e-μPAD was also applied for high-throughput determination of the concentrations of different metals, including Cd, Pb, Cu, Fe, and Ni metals, from a single sample of airborne particulate matter, and the results were in agreement with those obtained using a traditional method (95% confidence) [140].

**Figure 9:**
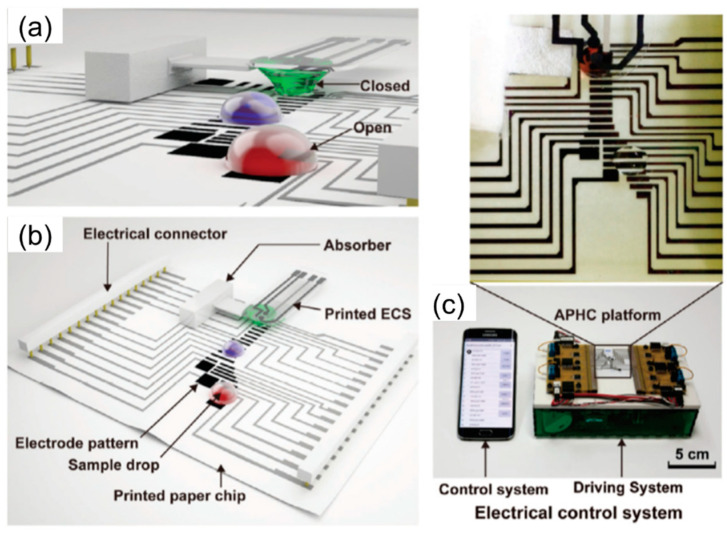
(**a**) Novel features of printed paper-based digital microfluidic chips include the partially open (blue and red drops) and closed (green drop) forms, (**b**) a modularly assembled active paper-based hybridized chip (APHC) platform, and (**c**) a combined wireless electrical control system with the chip platform, the driving system, and the mobile-based wireless control system. Reproduced with permission from the authors of [78]. Copyright 2017, WILEY-VCH Verlag GmbH & Co. KGaA, Weinheim.

Moreover, electrochemical detection seems well suited for integration with DMF technology because the processing steps required to form electrodes for droplet operation in the DMF devices are similar to those required to form electrodes for electrochemical detection. This combination represents a versatile system with high sensitivity, minimal reagent consumption, and automation capabilities for microscale analysis of many targeted analytes [141,142]. DMFs on paper substrates using electrochemical detection have become subjects of interest lately because the processes used to fabricate these devices are simple and incur lower costs. Ruecha et al. developed the first working electrochemical p-DMF sensors for multiplexed human healthcare monitoring by using printing and modular fabrication; these devices were operated using a portable, wireless control system. An electrochemical sensor and the electrodes on the device platform for electrowetting actuation of digital drops were fabricated using affordable printing techniques (Figure 9). The working electrode was modified with reduced graphene and Au NPs to enhance the sensitivity of the sensor. This hybridized open-close chip format demonstrated excellent multiplexed detection, which provided a wide linear range of low-level detection for three biological molecules in human serum [78]. During the fabrication step, a challenge for the design of the p-DMF device is the interference between the moving droplets and the voltages on the control lines because the electrode arrays and the control lines are printed on the same side of a paper sheet. To resolve this issue, researchers proposed control-fluidic codesign methodologies, which simultaneously adjusted the control line routing and the arrangement of the fluid droplets. The proposed method successfully eliminated the interferences during operation of the p-DMF device and achieved an optimal solution [143,144]. The improved droplet and electrode-oriented flows might increase the potential for p-DMF devices to be used as electrochemical detectors. All the publications reviews revealed the high potential for the use of electrochemical detection techniques with μPADs in the simultaneous detection of multiple analytes. Electrochemical techniques are open to further miniaturization, increased portability, and higher throughput ability for in-field use.

### 3.3. Chemiluminescence Detection

Chemiluminescence is an efficient analytical technique that has been successfully performed with μPADs by taking advantage of their simplicity and high sensitivity. For detection, this technique can reduce background noise without the need for an external light source, but a portable chemiluminescence reader must be operated in the dark, which makes device fabrication more complex. This detection method was used with μPADs for biomarker detection [145], immunoassay [146], biomolecule detection [147], environmental monitoring [148], and food safety control [149]. In recent years, chemiluminescence sensing has become popular for clinical biomarker detection and immunoassays due to its wide dynamic range and complete automation. Yang et al. demonstrated a novel paper-based chemiluminescence analytical device based on immunocomplex formation for the simultaneous detection of heart disease biomarkers. This device could be applied for simple, sensitive, and selective detection of three biomarkers in human serum and had low detection limits in the picogram range [145]. Guo et al. fabricated a μPAD by using a dipping method based on a chemiluminescence immunoreaction system for multiplexed detection of three cancer biomarkers in human serum samples [149]. Han et al. fabricated a novel paper-based immunoassay device for the chemiluminescence detection of allergen-specific immunoglobulin E (sIgE) in patients with allergy-related diseases. This strategy has led to a novel platform for use in clinical diagnosis [146].

**Figure 10:**
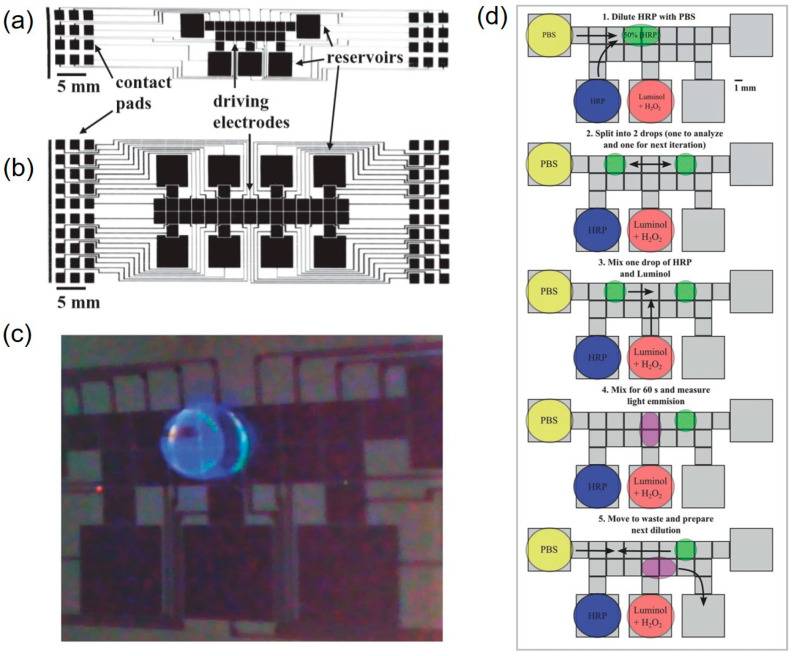
Photographs of p-DMF devices patterned with (**a**) Design A and (**b**) Design B. (**c**) Photograph of a device with its top plate removed for visualization, and (**d**) schematic illustration of the individual steps for homogeneous chemiluminescence assay using a p-DMF device. Reproduced with permission from the authors of [19]. Copyright 2014, WILEY-VCH Verlag GmbH & Co. KGaA, Weinheim.

Even though paper-based microfluidic concepts with chemiluminescence detection show great potential for the development of analytical devices with a variety of applications, these devices still have some drawbacks. In a paper-based format, assays, in particular ELISA, using such devices have low sensitivity and limited throughput capability. Therefore, p-DMF devices using chemiluminescence detection have been introduced to effect fully automated, complex, multistep assay protocols. Fobel et al. demonstrated for the first time a sandwich ELISA using chemiluminescence detection (Figure 10). The p-DMF device with more than one-hundred working parts was constructed by printing arrays of silver electrodes and reservoirs connected to contact pads onto a paper substrate. The proposed device was inexpensive, easy to use, and fast; one device cost less than $0.05 and could be fabricated in approximately 1 min. The device was applied for homogeneous, chemiluminescence ELISA of rubella IgG, which required 30 discrete steps for each concentration evaluated and showed a detection limit of 0.15 IU/mL [19]. This indicates that p-DMF devices based on inexpensive platforms can provide multiplexed performance and high detection sensitivity for chemiluminescence immunoassays. Overall, the chemiluminescence detection technique is providing new abilities for μPADs; however, the utility of chemiluminescence detection will depend on advances in cost reduction and increased simplicity of device fabrication.

### 3.4. Electrochemiluminescence Detection

**Figure 11:**
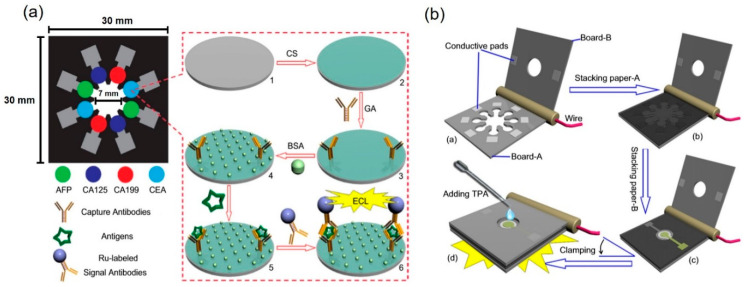
(**a**) Schematic representation of the platform features and assay procedures for this 3D electrochemiluminescence μPADs and (**b**) schematic representation of the facile homemade device holder. Reproduced with permission from the authors of [151]. Copyright 2011, Elsevier Ltd.

The electrochemiluminescence technique is based on electro-generated luminescence due to optical emission from an excited state of an electrochemiluminescent luminophore produced at the electrode’s surface by electrochemical reactions. The integration of electrochemical and spectroscopic methods provides better selectivity, increased dynamic concentration range, and multiplexed bioassay properties. Compared to chemiluminescence alone, this integrated technique stands out for its unique properties, such as lower background and greater control of the emission location and the reaction time. This facilitates the detection of multiple analytes and improves the reproducibility of an analytical process. Such μPADs have been used for multiplexed detection of electrochemiluminescence from biomarkers [150,151], immunoassays [152,153], biomolecules [154], and drugs [155]. To perform an electrochemiluminescence immunoassay with a μPAD for sensitive and multiplexed analysis, Ge et al. constructed a three-dimensional electrochemiluminescence μPAD based on wax-patterned technology and screen-printed electrodes for the detection of four tumor markers in serum samples (Figure 11) [151]. Electrochemiluminescence μPADs with bipolar electrodes have been used to promote both reduction and oxidation reactions without the need for an external power supply [153,154,156]. Zhang et al. developed a bipolar electrochemiluminescence paper-based platform consisting of three electrodes integrated into parallel into a single device for rapid, high-throughput detection of different biomarkers [153]. Liu et al. fabricated bipolar electrochemiluminescence μPADs in which multiple electrodes were situated in two parallel channels to achieve multiplexed detection of glucose in four complex samples [154].

Furthermore, the electrochemiluminescence technique has been integrated with DMF technology to achieve multiplexed assays with higher throughput. A DMF with electrochemiluminescence was demonstrated for the first time in 2017; that platform could move, dispense, mix, and split the droplets on arrays of insulated electrodes and was compatible with a wide range of analytical detection approaches. This novel platform was applied with a nucleic acid hybridization assay for the detection of miRNA and exhibited an analytical performance with a 1.5-fM detection limit for small sample volumes of 1.8 µL [157]. To our knowledge, no p-DMF devices using electrochemiluminescence detection have been reported so far. Nonetheless, the integration of electrochemiluminescence with DMFs has opened opportunities for p-DMF platforms, which offer lower costs, faster in-place fabrication with printers and ink, and better disposability. In the near future, fluid manipulation using DMF technology may be combined with capillary wetting features to create paper “hybrid” devices that take advantage of the significant capabilities of both formats for high-throughput electrochemiluminescence detection.

## 4. Applications of µPADs

As highlighted in the previous sections, μPADs are promising platforms that can be applied in various fields owing to their advantages of low cost, disposability, and ease of use when compared to traditional microfluidic devices [15,158,159]. The current applications of μPADs are summarized in Table 2. Due to the urge and importance of medical testing, μPADs development has focused more on medical diagnosis with high-throughput screening. Research has been conducted using real samples, such as human serum, blood, and urine, for the detection of multiple analytes. The common biomolecules studied in clinical diagnosis, including glucose [159], lactate [160], and uric acid [78], have been successfully identified by using μPADs with colorimetric sensing as the main technique for detection. To improve the throughput and the sensitivity of μPADs, researchers have developed and constructed p-DMF devices for use as diagnostic platforms for highly sensitive detection of multiple biomolecules [78,80,135].

### 4.1. µPADs for Nucleic Acid Amplification and Detection

Nucleic acids, including deoxyribonucleic acid (DNA) and ribonucleic acid (RNA), have long been interesting study subjects because of their importance in genetics, molecular analysis, detection, diagnosis, and monitoring. Nevertheless, nucleic acids exist in very small amounts in living cells: for example, only hundreds of nanograms of DNA can be extracted from one microliter of blood [161,162]). For that reason, the PCR and its modifications (quantitative PCR, reverse-transcriptase PCR, nested PCR, and multiplex PCR) are conventional methods used to amplify nucleic acids. Although these methods are very efficient, they often require special equipment and large amounts of laboratory space, consume significant amounts of power, and need to be applied by skilled, trained technicians. Since the explosion of microfluidic analytical devices, researchers around the world have directed their attention to the development and application of PCR chips as very promising goals. Various approaches based on distinct platforms from continuous-flow PCR chips to digital droplet PCR devices have been proposed, but at the time of this review, no work, to the best of our knowledge, has been reported for “paper-based PCRs”. This is reasonable because thermal cycles, especially at high temperatures, and the time-dependent continuous-flow of fluid are usually not recommended for paper or paper-related materials. This roadblock, however, has paved the way for the successful application of isothermal amplification techniques in µPADs for POCT. These techniques include, but are not limited to, nucleic acid sequence-based amplification (NASBA), transcription-mediated amplification (TMA), self-sustained sequence replication (3SR), helicase-dependent amplification (HDA), rolling-circle amplification (RCA), signal-mediated amplification of RNA technology (SMART), strand-displacement amplification (SDA), recombinase polymerase amplification (RPA), and loop-mediated isothermal amplification (LAMP), which is probably the most well-known of these techniques [163,164]. Generally, the above-mentioned techniques target specific and unique sequences of DNA [22,165] or RNA [166,167] and in many cases, microRNA (miRNA) [168,169,170]. Paper-based nucleic acid amplification and detection are useful in diseases diagnosis [22,23], forensic analysis [171], food [172] and beverage [173] control, and environmental testing [165]. Recently, several reviews on nucleic acid testing (NAT) using paper-based devices have been reported [174,175,176], so in this section, we only select and discuss those notable accomplishments that we believe have potential for, or may contribute significantly to, high-throughput screening.

As we discussed in the fabrication section of this review, µPADs for NAT should have integrated functions, as well as the capability to yield reproducible data, even though automation and mass production might still be limited. In this manner, Choi et al., for the first time, reported a paper-based biosensor that incorporated nucleic acid extraction, amplification, and visual detection or quantification by using a smartphone to detect *Escherichia coli* and *Streptococcus pneumonia* [173]. Their device consisted of four layers, including from top to bottom, (i) a lateral flow layer for visual detection, (ii) a glass fiber layer for immersion of nucleic acid, (iii) an FTA card for paper-based nucleic acid extraction, and (iv) a bottom layer for sample purification and washing. To control fluid flow between connecting layers, the authors used hydrophobic polyvinyl-chloride (PVC) backing pads as “valves”. The LAMP reaction was optimized at 65 °C for 45 min by using a handheld battery-powered heating device. Next, denaturation of double-strand DNA was performed at 95 °C for 30 s so that the product could be hybridized with the probe-labeled gold nanoparticles in the top layer. Finally, a lateral flow assay occurred in a disposable centrifuge tube. The entire “sample-to-answer” assay could be completed within 1 h. This system, however, suffered from the lack of both reproducibility and the capability to perform multiplex detection; furthermore, it was still complicated due to different temperatures that had to be applied.

Another effort came from Rodriguez et al., who provided a platform for molecular diagnosis of cervical cancer by combining nucleic acid isolation, isothermal amplification, and lateral flow assay for visual detection of human papillomavirus (HPV) 16 DNA [22]. The device contained (i) an adhesive base made from laminating sheets, (ii) a polyethersulfone (PES) filter paper disk for sample loading and nucleic acid extraction, (iii) an absorbent pad made from cellulose blotting paper for washing, and (iv) a lateral flow immunoassay (LFIA) detection strip, all assembled via folding and alignment. For testing, the clinical cervical specimen was initially resuspended in a lysis buffer, after which it was pipetted onto the sample port of the chip, where it wicked through the absorbent pad. After several washings, the absorbent pad was removed. Next, a LAMP reaction mix was added to the sample port, and another folding step was taken to seal and protect the sample from evaporation. The chip was placed on a 63 °C heating block or hot plate for 30 min for amplification. After incubation, part of the device was peeled off to expose the PES membrane to the LFIA. Once eluted by water, the amplified products could be introduced to the test strip, and results could be obtained within two minutes. Though innovative, this platform required many steps of complicated folding and fluid manipulation, which limited its usefulness for POCT. Later in 2018, the same group reported a similar, but simpler platform with the addition of internal amplification control for the detection of sexually transmitted infections caused by *Neisseria gonorrhoeae* [23].

To address multiplex NAT, in 2016, Cooper’s group published a notable study regarding the use of a paper-based origami device to diagnose malaria by using whole blood from finger prick [177]. Their device consisted of five panels fabricated by wax printing one folded onto another and a plastic cover for LAMP incubation. LAMP reagents were predeposited onto four different spots for amplification of different species; including spots for internal control, *Plasmodium pan, Plasmodium falciparum,* and *Plasmodium vivax*. The sample was added onto the folded panels, followed by the addition of lysis and a washing buffer for DNA extraction and purification. Next, the folded panel was turned over, and elution started to deliver purified DNA to the final panel for amplification and detection. LAMP reactions were carried out at 63 °C for 45 min on a simple hotplate, after which results could be simply readout using a handheld UV lamp. Later in 2019, the same group upgraded their device to a more user-friendly platform and applied it to POCT in low-resource rural communities in Uganda [86]. With the embedded finger-activated pumps for fluid transportation and the inclusion of lateral flow assay, the results could be easily read without the need for an additional readout device. Sixty-seven patients were tested, and the device provided an impressive sensitivity of 98% for *Plasmodium pan* and *Plasmodium falciparum*.

Considerable improvements in NAT using µPADs have been achieved. To avoid the relatively high temperature and long reaction time of LAMP, Margo et al. used reverse transcription-RPA for multiplexed detection of different RNA sequences of the Ebola virus [178], and Ahn et al. utilized RPA for simultaneous detection of multiple foodborne pathogens [172]. These isothermal reactions were complete 20 min after the injection of isolated RNA or DNA samples at temperatures ranging from 37 to 42 °C. Integrating this with miRNA extraction, Deng et al. employed the exponential amplification reaction (EXPAR), which allowed amplification at 37 °C in 20 min by using a portable heating block for detection of miR-21 and miR-155, two potential biomarkers associated with lung cancer [168]. To increase the sensitivity of fluorimetric detection of miRNA in µPAD, Liang et al. proposed a method of growing an interconnected, dense flower-like silver (FLS) layer on the surface of a cellulose fiber in the fluorescence detection zone [169]. This FLS-µPAD rendered not only a reduced background fluorescence signal but also a sensitive metal-enhanced fluorescence resonance-energy transfer (FRET) efficiency. On the other hand, for colorimetric detection, Teengam et al. promoted the use of a cationic pyrrolidinyl peptide nucleic acid (PNA) probe as a replacement for the DNA and the RNA probes [179]. The anionic silver nanoparticles (AgNPs) interacted with the cationic PNA probe and aggregated. If a complementary target sequence was present, it outcompeted and disaggregated the AgNPs, leading to a change of color in the detection zone. Albeit promising, if a convenient, all-in-one device is to be had, these tracks need to incorporate more functions.

To adapt to the new trend toward simpler, faster, but effective detection in NAT, Bender et al. presented a µPAD that engaged isotachophoresis (ITP) and RPA to simultaneously extract and amplify target nucleic acids [180]. The device consisted of a plasma separation membrane placed on top of a porous glass fiber strip, which was located between two liquid buffer reservoirs and was housed within a sealed Petri dish; two electrodes were embedded in the plastic lid for this system. A whole blood sample was pipetted onto the membrane where blood cells were held as the filtered plasma wicked into the proteinase pretreated sample pad region of the glass fiber strip. Next, a mixture of leading electrolyte (LE) and RPA reagents was added to the wet region from the glass fiber strip to the LE reservoir. Reservoirs were then filled with LE and trailing electrolyte (TE) solutions. Next, the chip was sealed by closing the lid. Once an electric field had been applied, nucleic acids were separated from the whole blood sample, and together with the RPA reagents, they gathered within an ITP plug where amplification took place; results could be observed with a fluorescence microscope. Interestingly, without the use of any specific heating device, the IPT-RPA still occurred by leveraging the Joule heating effect when a current was used to attain an appropriate temperature for RPA within the ITP plug. When the temperature was controlled using a hot plate, the total processing time was less than 20 min, including 5 min for filtration and digestion and 13 min for separation and amplification. Another creative idea came from Kaarj et al., who separated Zika virus RNA from different samples just by capillary action in a single-layer printed wax-paper channel [181]. Bulky biomolecules remained at the beginning of the channel while virus RNA could pass through and be focused at the end of the chip, where it was cut out and amplified by using an RT-LAMP reaction coupled with a pH indicator-based colorimetric assay. Although these systems still need further improvement, they have encouraged researchers to explore all possible fabrication and detection methods for NAT using a µPAD.

**Figure 12:**
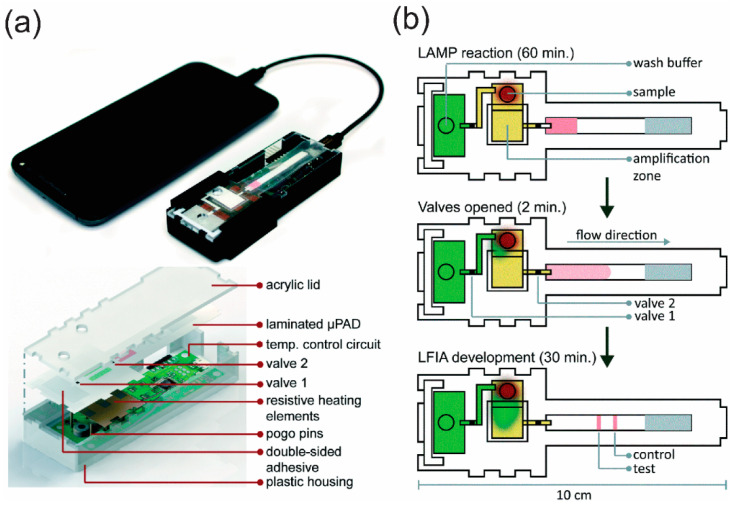
Microfluidic rapid and autonomous analytical device (microRAAD) to detect HIV from whole blood samples. (**a**) Photo of microRAAD connected to the phone to power the temperature control circuit and inner assembly of the device. (**b**) Schematic of fluid travel through microRAAD after sample deposition. Reproduced with permission from the authors of [182]. Copyright 2019, The Royal Society of Chemistry.

We finish by discussing a published work from Phillips et al. for a POC microfluidic rapid and autonomous analysis device (microRAAD) for the detection of the human immunodeficiency virus (HIV) [182]. The apparatus was built using a laminated and replaceable μPAD, a Kapton tape with printed resistive heating elements, and a temperature control circuit that could connect to a power source like a cell phone or portable battery, all assembled inside a plastic case as shown in Figure 12a. The main component, the μPAD, was composed of a glass fiber membrane for wash buffer distribution, a blood separator membrane (MF1), a PES filter membrane sandwiched between two dried polyethylene terephthalate (PEF) films preloaded with RT-LAMP reagents, wax valve strips for fluidic manipulation control, and an LFIA strip for visual detection. A whole blood sample was dropped into the sample inlet, followed by the addition of RT-LAMP rehydrating mixture, with a washing buffer being added into the buffer inlet. The two inlets were sealed with adhesive tape to avoid evaporation. Here, the wax valves acted as barriers to constrain flows from the buffer zone to the amplification zone and from the amplification zone to the LFIA. Fluid movements were illustrated in Figure 12b through three main steps: (i) Once powered, the amplification zone was heated first, reaching 65 °C within seconds and remaining at that temperature for 60 min. The pre-set temperature control circuit then automatically terminated heating in this zone and initiated heating of the two wax valves at the same time. (ii) Upon heating, the wax valves opened, and washing buffer was passed through the amplification zone, carrying amplicons, if any, to the LFIA. Hence, result bands could be observed 5 to 10 min after the valves had been opened (iii). In fact, to the best of our knowledge, this is the most complete system reported to date, yet it was not equipped with internal control and multiplex testing; furthermore, the performance time was also longer than those for other systems. Nevertheless, undoubtedly, these weaknesses can be overcome by careful adjustment and modification. This example, once again, points to a wide application of μPADs in NAT for POCT in the very near future.

### 4.2. µPADs for Detection of Bacterial Infection

In the diagnosis of diseases of interest, the use of sensitive, disposable μPADs will allow early diagnosis and greatly increase the chances for successful treatment. Several researchers have focused on the development of μPADs for multiplexed detection of cancer biomarkers and tumor cells based on the immunoassay method. These μPADs were mainly integrated with chemiluminescence [183] and electrochemiluminescence techniques [152]. For the immunoassay, some researchers have developed μPADs using the colorimetric method, but the use of those devices was often limited by insufficient sensitivity and a higher detection limit in comparison to other spectrometric methods. Thus, a p-DMF device using chemiluminescence detection has been proposed to enhance the detection sensitivity and the multiplexing capability of immunoassays [19]. Moreover, μPADs have been demonstrated to have considerable potential for POCT to help in the diagnosis of diseases caused by bacteria. Infectious bacterial diseases have always been problematic worldwide; infections arising from common bacteria, such as *Acinetobacter baumannii, Escherichia coli, and Staphylococcus aureu,* have increased drastically in recent years. Hence, the demand for a simple device with a fast response for the simultaneous screening of multiple bacteria is high. An aptamer μPAD for multiplex analysis of whole-cell bacteria based on aptamers bound to nitrocellulose membranes integrated into a chip has been developed. The detection involves a change in the color intensity as a bacterial cell attaches itself to specific aptamers labeled with biotin [184].

### 4.3. µPADs for Drug Screening

The capability of μPADs has been further expanded to drug analysis and screening in a high-throughput manner. A simple μPAD designed with a barcode feature has been utilized in the colorimetric detection of illegal drugs based on a flow lateral immunoassay system labeled with AuNPs [26]. A facile e-μPAD has been fabricated based on gold-paper screen-printed electrodes and the principle of Kirigami and origami. This μPAD was successfully used to electrochemically detect cancer cells and to screen in situ anticancer drugs in a high-throughput system with a low detection limit and wide linear range [185]. In the development of conventional µPADs for high-throughput drug analysis and screening, the devices need to be designed as multilayers by folding sheets of paper, which complicates the fabrication process. The use of p-DMF devices is an alternative simple and inexpensive approach to performing highly sensitive multiplexed drug analysis and screening.

### 4.4. µPADs for Environmental Monitoring

Together with the explosion and development of science and technology, activities of human beings, especially in manufacturing, construction, transportation, and agriculture, have caused global impacts on nature, which leads to retroacting on human beings. Daily, a large number of pollutants are released into the air, soil, and water, bringing about air pollution, food contamination, health problems and imbalance of ecosystem, etc. Accordingly, the application of µPAD in environmental analysis also receives a lot of attention. In this manner, readers are redirected to well-organized reviews by Meredith et al. [186] and Kung et al. [187]. Compared to other testing methods, environmental analysis seems not to be limited by a low amount of sample; however, it requires a dedicated study design and a careful sampling plan to collect representative samples from a vast heterogeneous area and preserve in proper condition to avoid sample degradation [188]. Different from bulky analysis systems, µPAD lightens the workload but still fulfills requirements of environmental analysis and monitoring. For example, heavy metals and pesticides should be given more consideration for priority control because of their high toxicity, relative stability, and easy accumulation in living organisms. Thus, devices that allow on-site detection and accurate monitoring of environmental conditions are needed. μPADs have been developed to improve the accuracy and performance for multiplexed colorimetric detection of pesticides [82] and heavy metals in the air [158] and rivers [189,190]. The devices developed thus far are portable and can be used as rapid on-site monitoring platforms for the detection of multiple heavy-metal ions; they have a wide linear range and a low detection limit down to the microgram level.

**Table 2:** Recent applications of μPADs for high-throughput analyses in clinical diagnostics, drug detection, screening, environmental monitoring, and food and beverage evaluation.

Analyte	Sample Type	Detection Method	Detection Limit	Ref.
Clinical diagnostics				
Glucose Dopamine Uric acid	Human serum	Electrochemical	0.05 mM 0.5 µM 5.0 µM	[78]
Ebola virus (three distinct RNA sequences)	Blood	Colorimetric	107 copies/µL	[178]
Helicobacter pylori (bacteria) Hepatitis B Human Immunoglobulin G.		Colorimetric	30 µg/mL 3 µg/mL 300 ng/mL	[191]
DNA targets	Blood	Colorimetric	10^2^ copies	[192]
Lactate Glucose Uric acid	Not specified	Colorimetric Electrochemical	190 µM 0.18 mM 0.11 mM	[160]
N. meningitides genomic DNA arrays	Not specified	Colorimetric	4.4 nM	[193]
Hydrogen peroxide Glucose	Serum Urine Wine	Electrochemiluminescence	0.041 mM 0.03 mM	[154]
Glucose	Not specified	Colorimetric	Not specified	[135]
Glucose Creatinine Uric acid	Urine	Electrochemical	0.12 mM 0.084 mM 0.012 mM	[138]
Carcinoembryonic antigens (CEA) Carcinoma antigen 125 (CA-125) Alpha-fetoprotein (AFP)	Human serum	Chemiluminescence	0.1 ng/mL	[183]
Carcinoembryonic antigen (CEA) Neuronspecific enolase (NSE)	Serum	Electrochemical	2 pg/mL 10 pg/mL	[136]
Copeptin, Heart-type fatty-acid-binding protein (h-FABP) Cardiac troponin I (cTnI)	Human serum	Chemiluminescence	0.40 pg/mL 0.32 pg/mL 0.50 pg/mL	[145]
Glucose	Urine	Electrochemical	0.03 mM	[137]
Norepinephrine (NE) Serotonin P-aminophenol (pAP)	Not specified	Electrochemical	1.2 µM 1.2 µM 0.38 µM	[139]
Copeptin Heart-type fatty-acid-binding protein (h-FABP), Cardiac troponin I (cTnI)	Human serum	Chemiluminescence	0.40 pg/mL 0.32 pg/mL 0.50 pg/mL	[145]
Acinetobacter baumannii Escherichia coli Staphylococcus aureus	Bacterial cells	Colorimetric	10^3^ CFU/µL 10^4^ CFU/µL 10^5^ CFU/µL	[184]
Carcinoembryonic antigen (CEA) Prostate-specific antigen (PSA)	Human serum	Electrochemiluminescence	0.07 ng/ML 0.03 ng/mL	[152]
Glucose Creatinine Uric acid	Urine	Electrochemical	0.12 mM 0.084 mM 0.012 mM	[138]
Glucose Lactate	Serum	Colorimetric	0.31 mM 0.29 mM	[159]
Drug analysis and screening				
Cycloheximide Etoposide Camptothecin	Cell apoptosis	Electrochemical	12.5 µM 12.5 µM 1.5 µM	[184]
Doxorubicin (DOX)	Anticancer drug	Colorimetric	4 µg/mL	[128]
Cocaine Morphine Methamphetamine	Blood	Colorimetric	37.5 ng/mL	[26]
Paracetamol (PAR) Caffeine (CAF) Ascorbic acid (AA)	Drug	Electrochemical	0.04 mM 0.22 mM 0.40 mM	[25]
Ascorbic acid Glucose Nitrite	Beverages	Colorimetric	1.47 µM 20 mM 0.06 mM	[15]
Environmental monitoring				
Co Cu Fe Mn Cr Ni	Airborne PM	Colorimetric	8.16 ng 45.84 ng 0.0186 ng 10.08 ng 0.0152 ng 80.40 ng	[158]
Ni(II) Cu(II) Cr(VI)	Lake water	Colorimetric	4.8 mg/L 1.6 mg/L 0.18 mg/L	[189]
Lead (Pb) Barium (Ba) Antimony (Sb) Zinc (Zn) Aluminum (Al) Iron (Fe) Magnesium (Mg)	Water	Colorimetric	250 ppm 250 ppm 250 ppm 25 ppm 250 ppm 400 ppm 250 ppm	[190]
Methyl paraoxon	Not specified	Colorimetric	10 µM	[82]
Food and beverage analysis				
Ascorbic acid Glucose Nitrite	Beverages	Colorimetric	1.47 µM 20 mM 0.06 mM	[15]
β agonist	Swine hair	Chemiluminescence	1.0 nM	[194]

## 5. Conclusions and Future Perspectives

To date, significant progress in the development of µPADs for high-throughput screening has been made, various platforms for a short time and multiplex assays can be used for multiple samples from different individuals. As for traditional p-CMF devices, they are limited by low sensitivity and by difficulty in enabling multistep analysis with automatic processes because p-CMF devices suffer from passive capillary wicking. To circumvent, researchers re-engineered those devices by incorporating methods to control the fluid transport in paper channels such as layer assembly, wax valve usage, or patterning hydrophobic and hydrophilic substrates inside channels. When fluid transport is programmed, complicated assay protocols can be accomplished automatically without the need for an external control system. However, the fabrication of controllable p-CMFs still needs further improvements and new breakthroughs because the devices are being applied more frequently for analytical applications. Meanwhile, p-DMF devices may become a promising tool for screening multiple analytes in a variety of applications. Because the p-DMF device is based on the EWOD technique, which can be programmed to manage the fluid flow better, multiple target analytes can be detected with high sensitivity by using a single- or multistep assay protocol. Although p-DMF devices have unique advantages in terms of fluid flow control, they still require a portable electrical switching system and a controlled software interface for the assay protocols, which make the p-DMF devices more expensive than conventional p-CMF devices. Therefore, the usefulness of p-DMF devices will depend on advances in reducing the costs of detection assays and fabrication processes. Overall, the development of μPADs with a lower cost of fabrication and controllable protocol is strongly recommended to explore this novel approach to high-throughput screening of any potential target analyte.

It is worth noting that high-throughput screening can only be obtained when the whole system is equipped with high-throughput detection and readout tools. For that reason, researchers have utilized and combined different methods to increase sensitivity, accuracy, and reproducibility of the results. To make POCT and on-field monitoring more affordable, interestingly, supporting devices gradually have been scaled down to minimal but still in well-functioned forms such as printed heater [182], portable fluorometer [195], mobile power supply [196], handheld Raman spectrometer [197], etc. This is indeed a very dramatic transformation that triggers the competition between manufacturers and allows small enterprises to enter the market, thus opening up the opportunity of commercialization of µPADs. Either in the form of accessories modifications or functional integration, the application of µPADs in high-throughput screening has been extending in various fields. The more widely µPADs are being used, the more innovations are to be made, which bring more benefits to the society, especially in low-income or under-developed countries.

As we have shown in the previous part, µPADs are most suited to on-site health screening with high-throughput capability. This plays an important role in early diagnosis, (isolation, if it is a must) and treatment to eliminate the spread of infectious or deadly diseases throughout the community, particularly when epidemic or pandemic is happening. Notably, biologists have alerted that harmful microbes have the ability to accumulate mutations and evolve to resist antibiotics and drugs. To detect a new type of microbe, new µPADs need to be developed. Therefore, it is necessary to optimize and set up a clear procedure for the fast prototyping of new µPADs. Even in normal conditions, quick and regular health screening also helps to build up personal medical records, which can be used for precision medicine. When readout methods like barcodes [36] or QR codes [198] are being used, results can be converted to digital information. In the vision of smart E-government, if a well-mannered procedure and collection can be established, such digital information can be encrypted and stored in a centralized cloud computing system managed by the government. Whenever necessary, medical experts can access the database and withdraw essential information (without personal information) to predict and evaluate the risk of a new disease in the population.

To sum up, the basic foundation of µPADs has been well set up for more than a decade, and the potential usage of µPADs is updated frequently. By targeting different applications, ideally, researchers can use various fabrication methods to alter different compartments and ultimately build up their platforms that meet the requirements of high-throughput screening and commercialization. For sample-to-answer purposes, the future of µPAD will be a fully integrated system with delicate design and user-friendly interface, providing a trustable result for POCT, all coming with a low cost and ready to be used in even resource-limited regions. It might not solely contain paper, but it should be either totally disposable or partially reusable. Very optimistically, the emerging and expanding of µPAD would play an important role not only in the field of analytical chemistry or microfluidic study but also in generally any side of life, via a process of detecting and solving, improving quality of life.

## Figures and Tables

**Table 1:** Recent fabrication of μPADs including paper-based continuous-flow microfluidic (p-CMF) and paper-based DMF (p-DMF) devices.

P-CMF Devices				
Method	Usage	Materials	Equipment	References
**Wax Printing**	Creating hydrophobic barriers	Wax	Wax printer; Hot plate	[32,33,34,35]
**Inkjet Printing**	Creating hydrophobic barriers, patterning electrodes, high-resolution, printing and conductive patterns	Alkyl ketene dimer (AKD)/UV-curable acrylate ink/hydrophobic sol–gel, polystyrene, etc.	Inkjet printer	[36,37,38]
**Photolithography**	Create channels by photoresist	Photoresist and developer, photomask	Photolithography equipment	[6,39,40]
**Flexographic Printing**	Creating hydrophobic barriers and layers on paper substrates	Polystyrene ink/PDMS ink	Roll-to-roll flexography units	[41,42]
**Plasma Treatment**	Creating 2D and 3D channels; surface modification (change of hydrophilicity)	AKD/ octadecyltrichlorosilane (OTS)/pentafluoroethane (PFE) monomer; metal mask	Vacuum plasma reactor	[43,44,45]
**Laser Treatment**	Cutting, engraving, inducing photopolymerization	Photopolymer (for the purpose of photopolymerization)	Laser cutting and engraving machine	[46,47,48]
**Wet Etching**	Creating hydrophobic barriers	Trimethoxy-octadecyl silane (TMOS) and NaOH solutions, paper mask	Hot plate or oven (for heating)	[49]
**Screen Printing**	Creating hydrophobic barriers and electrodes (for electrochemical detection)	Wax/polystyrene/conductive ink; screen stencil	Hot plate or oven (for heating)	[50,51]
**Cutting and Shaping**	Creating hydrophilic channels as designed	Adhesive sheet (for fixing)	Knife plotter/digital craft cutter	[52,53,54]
**Spraying**	Creating hydrophobic area	Mask, back supporting plate, hydrophobic coating material, permanent magnetic and screws (for fixing)	None	[55]
**Chemical Vapor Deposition**	Creating hydrophobic channels	Trichlorosilane	Vacuum chamber, heat block, hot plate	[56]
**Embossing**	Creating 3D open channels	Acrylonitrile butadiene styrene (ABS) dies, liquid ethanol, R^F^SiCl_3_	3D printer/laser cutting machine for fabrication of dies	[53,57]
**Stacking**	Creating 3D channels, flow control	Layers of 2D patterned paper, double-sided adhesive tape	None	[53,58]
**Origami Folding**	Creating 3D channels, flow control	Wax patterning panels, binding clip	Wax printer	[59,60,61,62,63,64]
**3D Printing**	Creating 3D channels	Paraffin wax/photocurable resin	3D printer with a custom-made extruder	[65,66,67,68,69,70,71]
**Pen Writing**	Creating fluid barriers	Acrylate-based resin, titanium dioxide, permanent marker ink	3D pen, correction pen,	[72,73,74]
**Digital Plotting**	Creating fluid barriers	PDMS, permanent marker ink		[75,76,77]
**P-DMF Devices**				
**Inkjet Printing/Chemical Vapor Deposition** **(CVD)**	Patterning electrodes/creating dielectric layer	AgNP; CNT/parylene-C-Teflon	Inkjet printer/vapor deposition chamber; spin coater	[19,78]
**Ballpoint Pen Printing/Wrapping**	Patterning electrodes/creating dielectric layer	AgNP/plastic wrap–silicon oil	Conductive ballpoint pen; plotter	[79]
**Screen Printing/Taping**	Patterning electrodes/creating dielectric	Carbon; silver/adhesive tape-nevosil	Screen print	[80,81]
**Microsyringe Dispensing/Spin Coating**	Patterning electrodes/creating dielectric layer	AgNP/ PDMSsilicon oil	Microsyringe dispenser/spin coater	[82]
**Spraying/Taping**	Patterning electrodes/creating dielectric layer	Graphite/tape-nevosil	Spray	[83]
